# Engineering of hybrid anticancer drug-loaded polymeric nanoparticles delivery system for the treatment and care of lung cancer therapy

**DOI:** 10.1080/10717544.2021.1934187

**Published:** 2021-07-20

**Authors:** Yang Zhao, Kefeng Liu, Jie Li, Juan Liao, Li Ma

**Affiliations:** a Department of Pharmacy, The Fifth Affiliated Hospital of Zhengzhou University, Zhengzhou, PR China; b Department of Pharmacy, The First Affiliated Hospital of Zhengzhou University, Zhengzhou, PR China; c Third ward of Radiotherapy, Shaanxi Provincial Cancer Hospital, Xi’an, PR China; d Department of Medical Oncology, Beijing Tuberculosis and Thoracic Tumor Research Institute, Beijing Chest Hospital, Capital Medical University, Beijing, PR China

**Keywords:** Drug delivery, human lung cancer, cytotoxicity, apoptosis, hemolysis, nursing care

## Abstract

Chemotherapy with combination drugs has become one of the most commonly used cancer prevention treatments, with positive clinical results. The goal of this study was to develop compostable polymeric nanomaterials (NMs) for the delivery of puerarin (PRN) and 5-fluorouracil (5FU), as well as to investigate the anticancer activity of the drug delivery system (PRN-5FU NMs) against *in vitro* and *in vivo* lung cancer cells. Since double antitumor drugs PRN and 5FU are insufficiently compressed in polymer-based bio-degradable nanoparticles, encapsulation of PRN and 5FU antitumor drugs were co-encapsulated with polyethylene glycol and polylactidecoglycolide nanoparticles (NMs) is efficient. The arrangement of PRN NMs, 5FU NMs, and PRN-5FU NMs, as well as the nanoparticles shape and scale, were studied using transmission electron microscopy (TEM). 5FU-PRN NMs triggered apoptosis in lung carcinoma cell lines such as HEL-299 and A549 *in vitro*. Acridine orange/ethidium bromide (AO/EB) and nuclear damaging staining techniques were used to observe morphologies and cell death. The mechanistic analysis of apoptosis was also confirmed by flow cytometry analysis using dual staining. When compared to free anticancer products, the hemolysis analysis findings of the 5FU-PRN NMs showed excellent biocompatibility. Taken together the advantages, this combination drug conveyance strategy exposed that 5FU-PRN NMs could have a significant promising to improve the effectiveness of lung cancer cells.

## 1. Introduction

Combination treatment with an activator is partly owing to the combination of a promising potential anticancer drugs. The dosage proportion of two drugs, as well as the potential competitive molecules, decide the composition of an effective antitumor drug delivery system (Liang & Kiick, 2016; Brus et al., 2017; Sun et al., 2021; Wolfe et al., 2021). As a result, the prominence of maintaining a beneficial association in order to continue compatibility between the various components by biodegradable nanoparticle delivery cannot be overstated. Owing to the antitumor drugs' different physicochemical properties, encapsulating multiple anticancer medicines in a single nanoparticle has proved difficult (Yu et al., 2014; Tu et al., 2017; Rao et al., 2018; Xu et al., 2018). Bio-degradable nanoparticles, which are created by en5FUulating a variety of medications with discrete physico-chemical behaviors and controlling their controlled releasing in regulated proportions, are thus thought to be more effective at delivering drugs to cancer cells (Lee & Nguyen, 2013; Li et al., 2014; Dong et al., 2021). Hybrid anticancer delivery may be used to achieve combination treatment with a sensitizer (Mao et al., 2021; Pragya et al., 2021; Tran et al., 2021). The composition of a promising antitumor drug delivery mechanism is determined by the dosage ratio of the two drugs, as well as the possible rival molecules. As a result, the significance of creating a beneficial relationship in order to preserve synergy between the different substances through conveying nanoparticles cannot be overstated (Jain et al., 2010; Xu et al., 2016; Esmaeili et al., 2021). Encapsulating several anticancer medications in a single nanoparticle has proven difficult due to the antitumor drugs’ distinct physicochemical properties (Rayamajhi et al., 2019; Long et al., 2020; Nguyen et al., 2020; Wang et al., 2020; Sameiyan et al., 2021).

For the past 70 decades, fluoropyrimidine 5-fluorouracil (5FU) has been widely utilized as a frontline medication for the treatment of a wide variety of tumors, including colon, cervical, lung, and breast cancers. When given intravenously, it is aqueous soluble and stable (De Luca et al., 2016; Ai et al., 2019; Dai et al., 2020; Moorkoth et al., 2021). However, for early colorectal cancer, 5FU has a response rate ∼15%, and its biocompatibility decreases life times. 5FU also has an opposing effect on the gastrointestinal tract, as well as hematological, neurological, cardiovascular, and breast cancer treatments. Furthermore, a good drug delivery mechanism for 5FU must be established in order to attain greater treatment efficiency with less adverse effects. Due to non-target specific connections with various pharmacological and activation in both non-cancerous and cancer tissues, 5FU treatment reduced cell proliferation and triggered unfavorable responses (Rayamajhi et al., 2019; Long et al., 2020; Wang et al., 2020). As a result, more consistent and efficient 5FU therapy is needed. Nanomaterials (NMs) made of polymeric materials have been originate to be robust mechanisms for future therapeutic uses due to the large-scale processing (Lopes et al., 2012; Li et al., 2018; Huang et al., 2019).

Owing to its ecofriendly accountability and bio-competitiveness, bio-degradable polymeric nanoparticles with appropriate dimensions, even surface diameter, and increased aqueous solubility have gotten a lot of attention (Liu et al., 2016; Zhang & Tung, 2017; Liu et al., 2019; Li et al., 2020a, 2020b). In anticancer therapy, biomaterial fields, and medical diagnosis, a number of environmentally friendly NMs have been widely used. Biodegradable NMs can increase the therapeutic benefit of a variety of pharmaceutical drugs and bioactivities by increasing bioavailability, aqueous solubility, and retention times (Ambrogio et al., 2013; Song et al., 2017; Zhang et al., 2017; Wlodarczyk et al., 2018; Wu et al., 2020). Customers and businesses should have a limited supply of biodegradable NMs manufactured from nontoxic and recycled materials on hand. The manufacture of polymer composites for nanoparticles assembly is the subject of current biodegradable NMs research (Wang et al., 2019).

With all of the advantages in mind, we looked at the various factors that go into developing a nanoprecipitation method for combining two promising anticancer drugs into block-polymers (puerarin [PRN]/5FU NMs). The MTT assay was also used to investigate the proliferations of dual drugs co-loaded with nanoparticles for the treatment of lung cancer cells by *in vitro*. We analyzed the morphological characteristics of control cells and the treatment population using double staining approaches (acridine orange/ethidium bromide [AO/EB]). Flow cytometry analysis was used to examine the cell death in lung carcinoma cell lines.

## 2. Experimental section

### 2.1. Materials

Meilun Biotechnology Co., Ltd. obtained PRN and 5FU (Dalian, China). Xi'an Ruixi Biological Technology Co., Ltd. (Xi'an, China) given MTT, dimethyl sulfoxide (DMSO), Hoechst 33258, AO/EB, Annexin V-fluorescein isothiocyanate (FITC)/propidium iodide (PI) apoptosis detection package. Other reagents were obtained and used as received from Sinopharm Chemical Reagent Co., Ltd. (Shanghai, China).

### 2.2. Encapsulation of puerarin (PRN) and 5-fluorouracil (5FU) in 5FU-PRN NMs

PRN and 5FU encapsulation in PEG-PLGA was improved using the evaporations technique for oil/water solvent method. In a nutshell, PRN (100 g) and 5FU (100 g) coated with dioleoyl phosphatidylserine acid (DOPA) are combined with PLGA-NMs formulations in CHCl_3_ (50 mL). A 5 mL PBS solution was mixed with a 10% PVA solution formulated with CHCl_3_. The results were mixed for 12 h, and the water components were vaporized. For potential usage, PEG-PLGA NMs (PRN-5FU NMs) were prepared with 5FU and PRN and deposited at −20 °C. To evaporate dual emulsification solutions and achieve an oil/water composition, PEG-PLGA NMs surface coated by DOPA-covered 5FU and PRN is used. The polymeric nanoemulsions were immersed with PEG-PLGA solution to vaporize the solvent substances. The aqueous formulations were vaporized after 12 h of incubation.

### 2.3. In vitro *dual drug release profiles*

Diffusions dialysis was used to assess the *in vitro* release of two drugs. Solutions equivalent to 5FU-PRN NMs (0.1 mg/mL), 5FU, and PRN (0.25 mg/mL) were obtained from the dialysis disguise. These NMs were discrete in 25 mL of released mediums formulated in different pH 5 and 7.4 by PBS with 0.2% Tween-80 solution, while the regulated releasing fresh medium was extracted from the deviancy shaker at 1500 RPM in 37 °C and an equal volume of complete new mediums was used. The UV spectrometer was used to examine the controlled drug release effects of 5FU and PRN, as previously mentioned (Wilems et al., 2015; Stein et al., 2018; Kang et al., 2019; Li et al., 2020a, 2020b).

### 2.4. *Examination of* in vitro *proliferations*

HEL-299 and A549 human cancer cell lines were grown in Dulbecco’s modified Eagle’s medium (DMEM, Gibco, Grand Island, NE, which contained 10% fetal bovine serum (FBS) and 1% penicillin-streptomycin. In a humidified incubator, cells were held at 37 °C with 5% CO_2_.

### 2.5. Examination of apoptotic staining methods

A molecular shrinkages examination utilizing AO/EB and nuclear staining was achieved to evaluate the morphology features in the HEL-299 and A549 cells. After 24 h of treatment, the HEL-299 and A549 cells were grown in 24 well plates at a concentration of 1 × 10^4^ cells per well. The same plate was treated with 5FU-PRN NMs at IC_50_ concentrations. The next day, both the treated and control cell lines were stained with dual staining substances. The 48 well plate was then washed three times with 1X PBS as a result. Photos of cancer cells were captured using fluorescence microscopy (CLSM, Laser TCS SP5 II, Germany) (Mohamed Subarkhan et al., 2016; Subarkhan & Ramesh, 2016; Balaji et al., 2020; Sathiya Kamatchi et al., 2020).

### 2.6. Examination of cell death

The rate of apoptosis was determined using the Annexin V-FITC/PI Apoptosis Detection Kit. HEL-299 and A549 cells were seeded at a density of 5 × 103 cells/well in 6-well microplates with DMEM for 24 h at 37 °C and 5% CO_2_, and then treated with 5FU NMs, PRN NMs, and 5FU-PRN NMs at IC_50_ concentrations for 24 h at 37 °C and 5% CO_2_. Cells were collected and washed once in PBS before being incubated in 195 µL binding buffer (1X) and 5 µL Annexin V-FITC. After a 5-min centrifugation at 1500 g at 4 °C, each tube was washed with 200 µL binding buffer (1X); 190 µL binding buffer (1X) and 10 µL PI solution were applied to resuspended the cells, and the tubes were incubated at room temperature for 15 min. The rate of cell apoptosis was calculated using an FC500 flow cytometer (Beckman Coulter, Inc., Brea, CA), and the data were analyzed using FCS analysis software (Mohamed Subarkhan et al., 2019; Subarkhan & Ramesh, 2016; Mohan et al., 2018).

### 2.7. Hemolysis assay

The Department of Medical Oncology, Beijing Tuberculosis and Thoracic Tumor Research Institute, Beijing Chest Hospital, Capital Medical University, Beijing-101149, China were approved for collection of new human blood samples. Samples of newly collected human blood were taken. In the previous methods, we performed hemolysis (Fischer et al., 2003; Tramer et al., 2012; Evans et al., 2013; Pham et al., 2014). The blood was centrifuged, the solutions were extracted, cold PBS cleaned, and blood removed completely three times and the HRBCs were obtained. Subsequently, cold PBS diluted HRBCs (0.1) solutions. With 0.9 mL of DD-water, the solution was transferred to the 5 ml tubes and used in positive regulation. Often used as a negative control are 0.9 mL. In addition, the PBS contains GEM and GEM-NP solutions (5–30 μg/mL). The mixture was incubated in 3 h later and the centrifuge continued and the absorption was measured by using the general formula using the UV-spectrometer. The hemolysis percentage = (As – An)/(Ap – An) = 100%. Where As, An and Ap are the absorption and negative regulation of the sample, respectively, the positive control.

### 2.8. Statistical analysis

The mean standard deviation was used to present quantitative results. The magnitude of differences between two groups was determined using the Student’s *t* test, while differences between more than two groups were determined using one-way ANOVA using a Turkey’s *post hoc* test. A *p<.05 value was found statistically important.

## 3. Results and discussion

### 3.1. Nanoparticles construction and characterization

**Figure 1.**
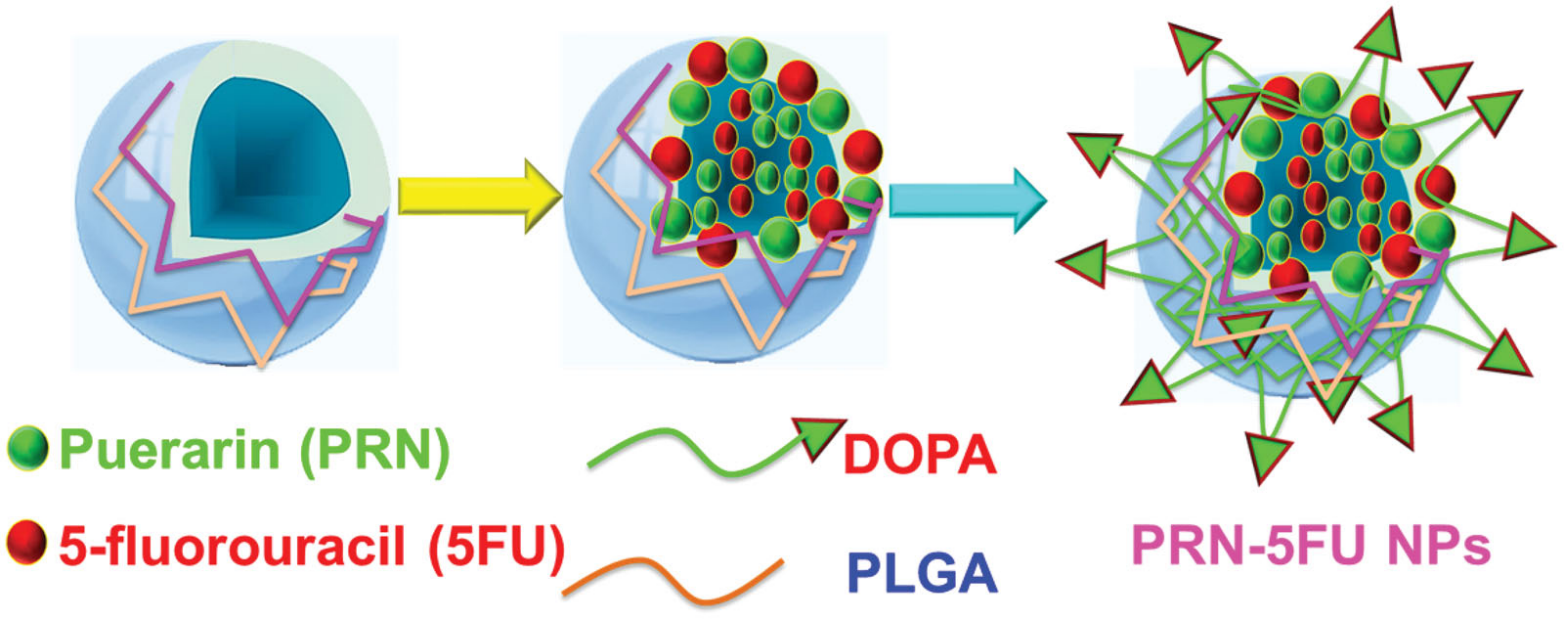
DOPA-coated PRN and 5FU facilitated the self-assembly of polymer nanoparticles (PRN-5FU NMs) to enhance the in vitro impact on lung cancer cell lines.

The ability to deliver dual combination therapy is enabled by the growth of capably stacked 5FU and PRN encapsulated on polymeric nanoparticles (known as PRN-5FU NMs). 5FU and PRN, for example, are hydrophobic molecules that can be combined to form PRN-5FU NMs. The PRN test was chosen because of its utility for 5FU, and its centers were inserted into 5FU-PRN NMs near 5FU. Figure 1 depicts the main stacking structures of PRN and 5FU within PRN-5FU NMs. The 5FU-PRN NMs hydrophobic polymer interaction structure included PRN and 5FU. Dual-polymeric segments, as well as similarities to PRN and 5FU. Polymeric nanoparticles (PRN-5FU NMs) were formed quickly with 5FU at 10 mg/mL and PRN at 10 mg/mL to create lipid-soluble molecular frameworks, as exposed in Figure 1.

**Figure 2.**
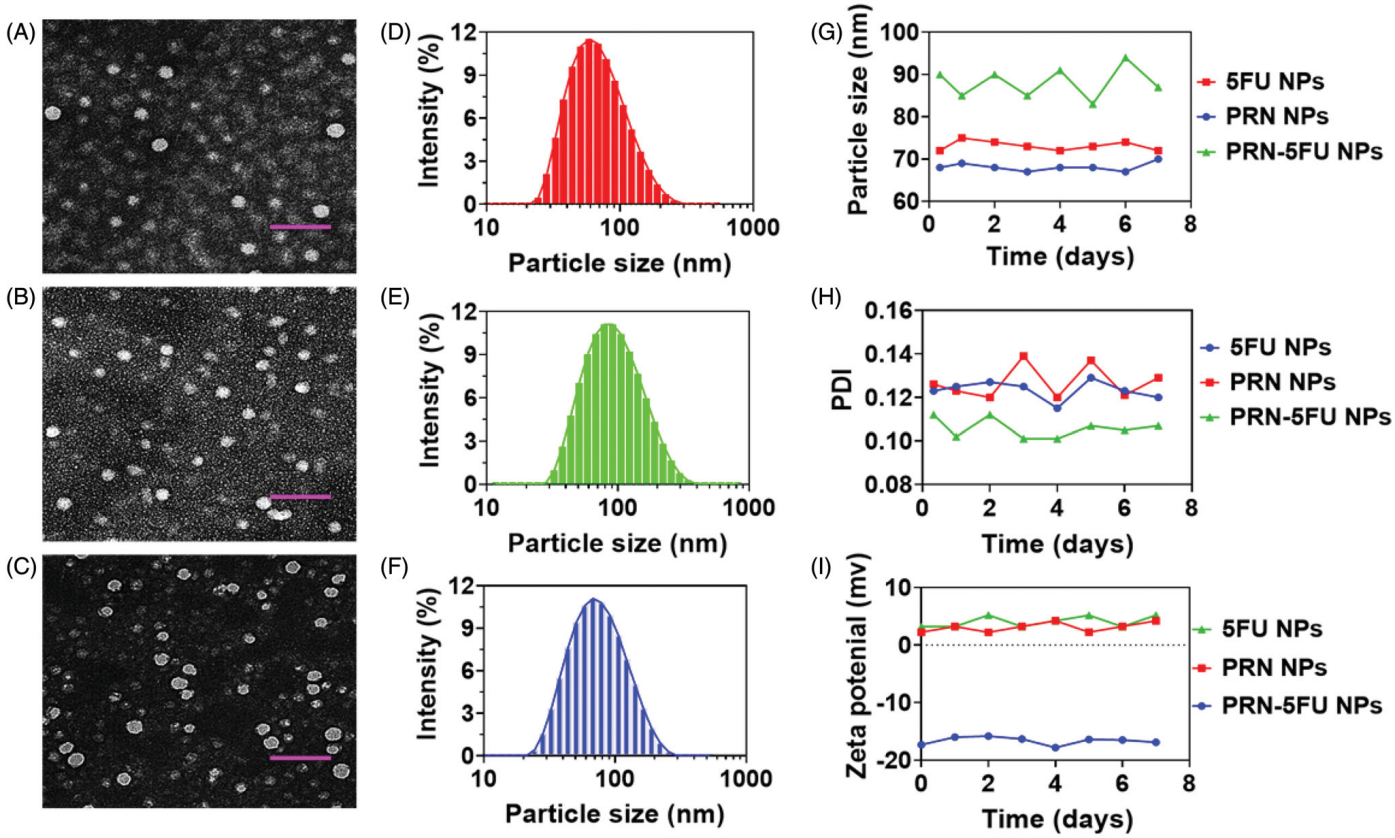
The morphological characteristics of 5FU NMs, PRN NMs, and PRN-5FU NMs. (A–C) TEM images of various self-assembled nanoparticles (5FU NMs, PRN NMs, and PRN-5FU NMs). Scale bar 200 nm. (D–F) The size distributions of 5FU NMs, PRN NMs, and 5FU-PRN NMs assessed using DLS methods. (G–I) The stability of nanoparticles (5FU NMs, PRN NMs, and PRN-5FU NMs) for 7 d post formulation assessed using DLS technique.

The impact of 5FU and PRN on lung carcinoma therapy was assessed in this examination, which included PRN/5FU@NP. The resulting NMs were subjected to TEM examinations in order to conduct a basic design analysis. TEM analysis displayed the influence of the morphology properties of 5FU NMs, PRN NMs, and PRN-5FU NMs. 5FU-PRN NMs were successfully collected, as shown by the results in Figures 2(A–C). The morphology of the synthesized nanoparticles was also investigated using HR-TEM. The nanoparticles were extracted using clusters of emulsified hydroxyapatite nanoparticles (Figure 2(A–C)), and the size of 5FU-PRN NMs was calculated using dynamic light scattering (DLS). The particles sizes of 5FU NMs, PRN NMs, and 5FU-PRN NMs were monitored by utilizing the TEM image between 72.4 ± 0.7, 71.4 ± 0.3, and 81.5 ± 0.6 nm (Figure 2(D–F)), respectively, and the polydispersity index is between 0.148 ± 0.04, 0.181 ± 0.03, and 0.157 ± 0.04, which corresponds with the outcomes of the DLS measurements, and, therefore, deliver durable validation of the NMs constancy of 5FU NMs, PRN NMs, and 5FU-PRN NMs in PBS conditions was investigated using complex dispersions of particle diameters of 5FU NMs, PRN NMs, and PRN-5FU NMs. The polydispersive indices, precisely for 5FU NMs, PRN NMs, and PRN-5FU NMs, were measured at a ratio of 100:1, and then incubated for 30 min at 37 °C to further investigate the nanoparticles (Figure 2(G–H)). Furthermore, DLS measurements revealed that the zeta potential and constancy of 5FU NMs, PRN NMs, and 5FU-PRN NMs were 4.2 ± 0.2, 7.5 ± 0.3, and −7.8 ± 0.3 mV, respectively (Figure 2(I)).

### 3.2. *In vitro* dual drug release profile

**Figure 3.**
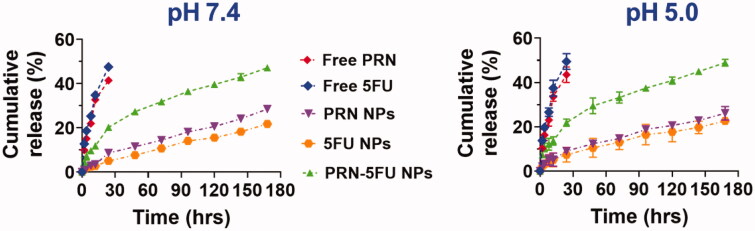
*In vitro* release profiles of 5FU and PRN from 5FU NMs, PRN NMs, and 5FU-PRN NMs at 37 °C with pH 5 and 7.4.

The miseries, drug loading capability, sizes, and aqueous solubility of the resulting nanoparticle frameworks are all determined by the release profiles of 5FU-PRN NMs (Fischer et al., 2003; Pham et al., 2014; Subarkhan & Ramesh, 2016; Mohan et al., 2018). The drug release rate of 5FU-PRN NMs loaded with 5FU and PRN reverted, resulting in improved matrix efficiency, according to the findings. Uncontrolled release, on the other hand, occurs regardless of whether the drugs are folded, open, or unchecked. These techniques reveal how shell holes shape, allowing drugs to be released in a controlled manner. The kinetic mediated controlled release is verified using a physico-chemical examination of 5FU-PRN NMs and drug delivery. The results of sustained PRN-5FU NMs, 5FU, and PRN drug delivery were studied using this dialysis technique. A constant release analysis was carried out in PBS medium at 37 °C and pH 5 and 7.4. The 5FU-PRN NMs were overwhelmed by the controls release profiles definitions of 5FU and PRN variants, implying that the initial release took about 5 h, followed by a 6-d slow release (Figure 3). At the beginning of the 10 h following the formation of PRN-5FU NMs, half of the 5FU and PRN were clear. At different pH values, no major differences in 5FU and PRN release from 5FU-PRN NMs were observed during the study. As a result, 24 h later, a moderate release of 40–50% was observed. The charging of 5FU and PRN on the substrate of 5FU-PRN NMs had no negative effects on the supervised release of these NMs, according to these results.

### 3.3. Examination of *in vitro* proliferations

**Figure 4.**
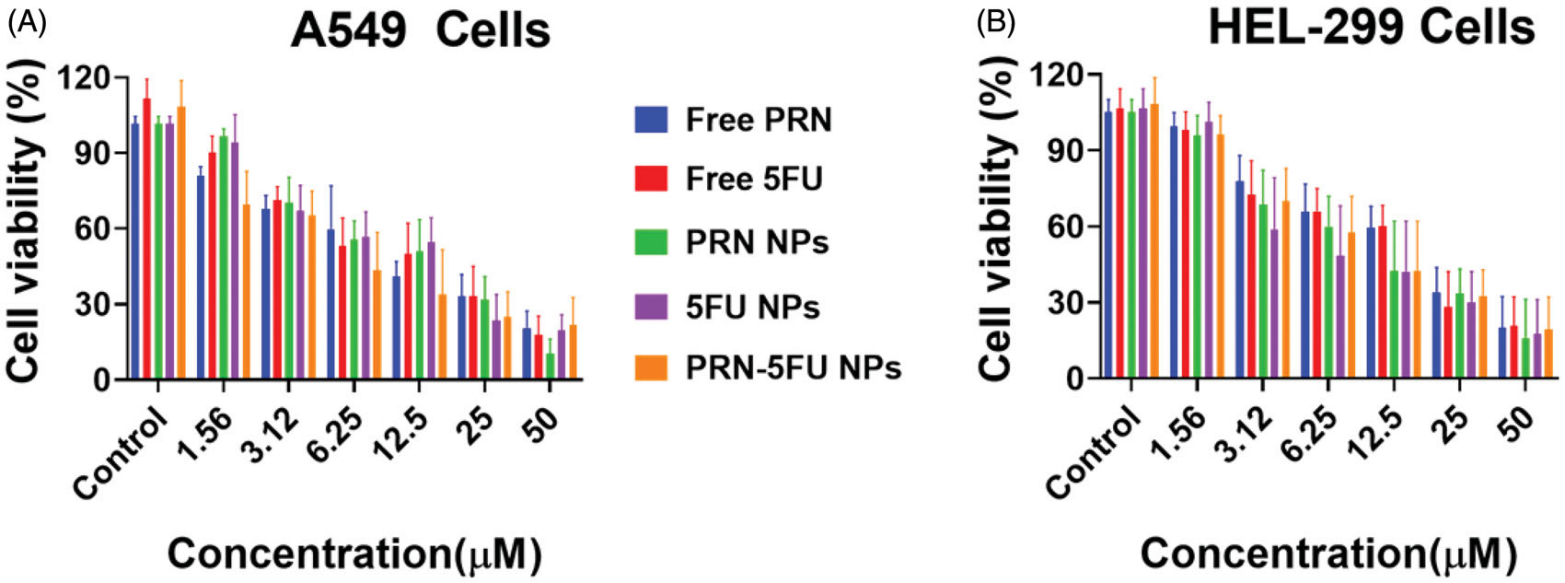
*In vitro* proliferation outcomes of the synthesized 5FU NMs, PRN NMs, and PRN-5FU NMs. A-B) Cell proliferation of human lung carcinoma cells (HEL-299 and A549) after the treatment with 5FU NMs, PRN NMs, and 5FU-PRN NMs for 24 h. (C) Cell proliferation of HUVEC non-cancer cells following the treatment with different nanoparticles for 24 h.

MTT investigation was conducted out after the effective and active fabrication of 5FU-PRN NMs to determine the anti-tumor property of free 5FU, free PRN, 5FU NMs, PRN NMs, and 5FU-PRN NMs on lung cells such as HEL-299 and A549. Cells proliferations was observed after 24 h of treatments, and dose-based curve exploration revealed the inhibition activity (5FU NMs, PRN NMs, and PRN-5FU NMs) (Figure 4(A,B)). 5FU-PRN NMs had cytotoxic effects on human lung carcinoma cells, which was surprising. The IC_50_ values for free 5FU, free PRN, 5FU NMs, PRN NMs, and 5FU-PRN NMs in the A549cell line were 13.04 ± 2.54, 14.45 ± 2.47, 10.24 ± 3.14, 9.54 ± 1.48, and 3.98 ± 2.14, respectively. The IC_50_ values for free 5FU, free PRN, 5FU NMs, PRN NMs, and 5FU-PRN NMs in HEL-299 cell lines were 19.47 ± 3.24, 20.14 ± 2.47, 10.57 ± 3.28, 11.24 ± 3.24, and 5.12 ± 2.45, respectively. The proliferation action of 5FU-PRN NMs was increased when chemotherapeutic agents were delivered simultaneously into cancer cells. Furthermore, the hydrophilic PLGA segments form a water soluble polymeric membranes coating that enhances the accessibility of cellular membranes via the bilayer membrane.

### 3.4. Examination morphology features (AO/EB)

**Figure 5.**
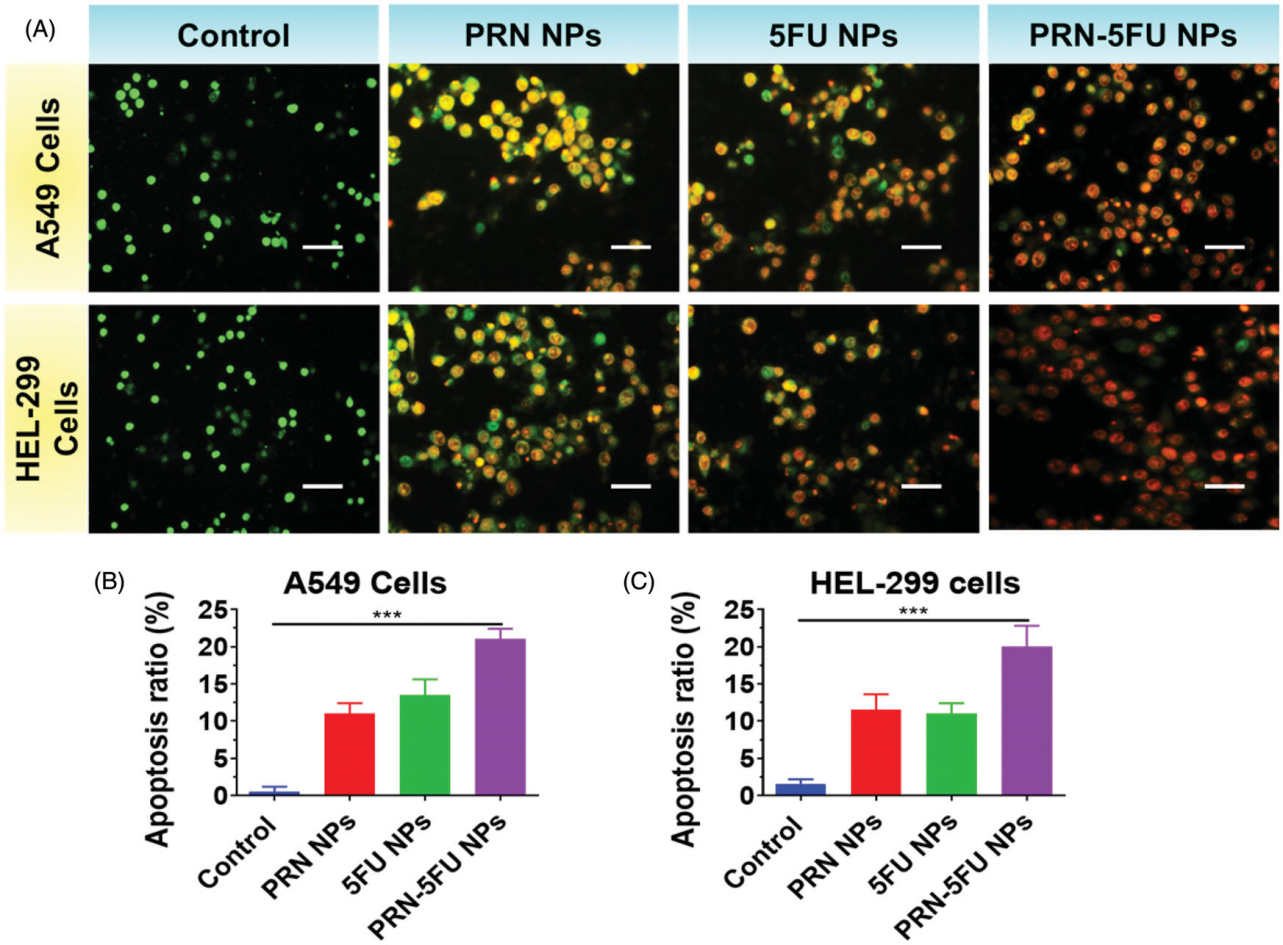
Assessment of apoptosis in human lung carcinoma cells (HEL-299 and A549) were treated with synthesized nanoparticles. HEL-299 and A549 cells incubated at 37 °C for 24 h with different nanoparticles (control, 5FU NMs, PRN NMs, and PRN-5FU NMs) for 24 h. The living cells are stained in green by AO, whereas the dead cells are stained in red by EB. The scale bar represents 20 μM.

An optical inverted phase contrast microscope was used to examine the morphological features of HEL-299 and A549 cells to see whether they changed after being exposed to 5FU NMs, PRN NMs, and PRN-5FU NMs. Figure 5(A–C) shows that after treatments with 5FU NMs, PRN NMs, and PRN-5FU NMs, HEL-299 and A549 cells were smaller and more rounded than the control group.

### 3.5. Examination morphology features (Hoechst 33258)

**Figure 6.**
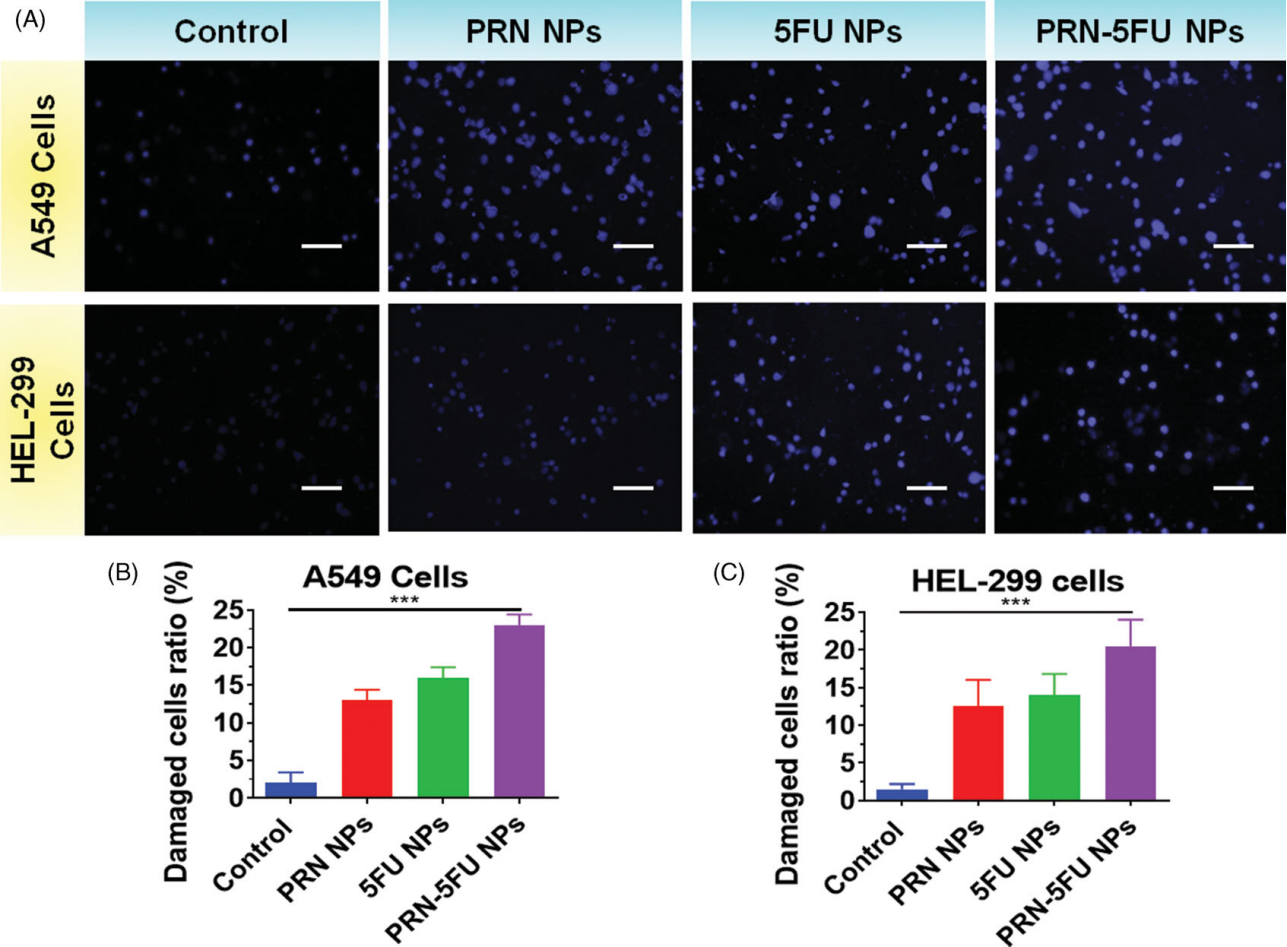
Assessment of apoptosis in human lung carcinoma cells (HEL-299 and A549) were treated with synthesized nanoparticles. HEL-299 and A549 cells were incubated at 37 °C for 24 h with separate samples (control, 5FU NMs, PRN NMs, and PRN-5FU NMs) for 24 h. The nucleus was discolored with Hoechst 33342 staining. The scale bar represents 20 μM.

After Hoechst 33258 nuclear staining, 5FU NMs, PRN NMs, and PRN-5FU NMs-treated cells displayed brighter blue light than control cells, suggesting pyknotic and deep-dyed nuclei. Some of the 5FU NMs, PRN NMs, and PRN-5FU NMs-treated HEL-299 and A549 cells released orange and red fluorescence that was smaller in size than the control group, according to AO/EB staining (Figure 6(A–C)).

Cell morphologies were stained with AO/EB and Hoechst 33258 to confirm the effect of 5FU NMs, PRN NMs, and 5FU-PRN NMs on HEL-299 and A549 cells by inducing apoptosis. AO/EB staining can distinguish between apoptotic and necrotic cells due to differences in fluorescence. Figure 5 shows the control group, which had normal morphology and pale green fluorescence. The number of cells gradually decreased as cells were exposed to varying quantities of 5FU NMs, PRN NMs, and PRN-5FU NMs, and some cells emitted bright green and red fluorescence. When HEL-299 and A549 cells were stained with Hoechst 33258 after being treated with 5FU NMs, PRN NMs, and PRN-5FU NMs, typical apoptotic features such as chromatin condensation and disruptive nuclei fragmentation were observed, as shown in Figures 5 and 6.

### 3.6. Evaluation of cell death mechanism

**Figure 7.**
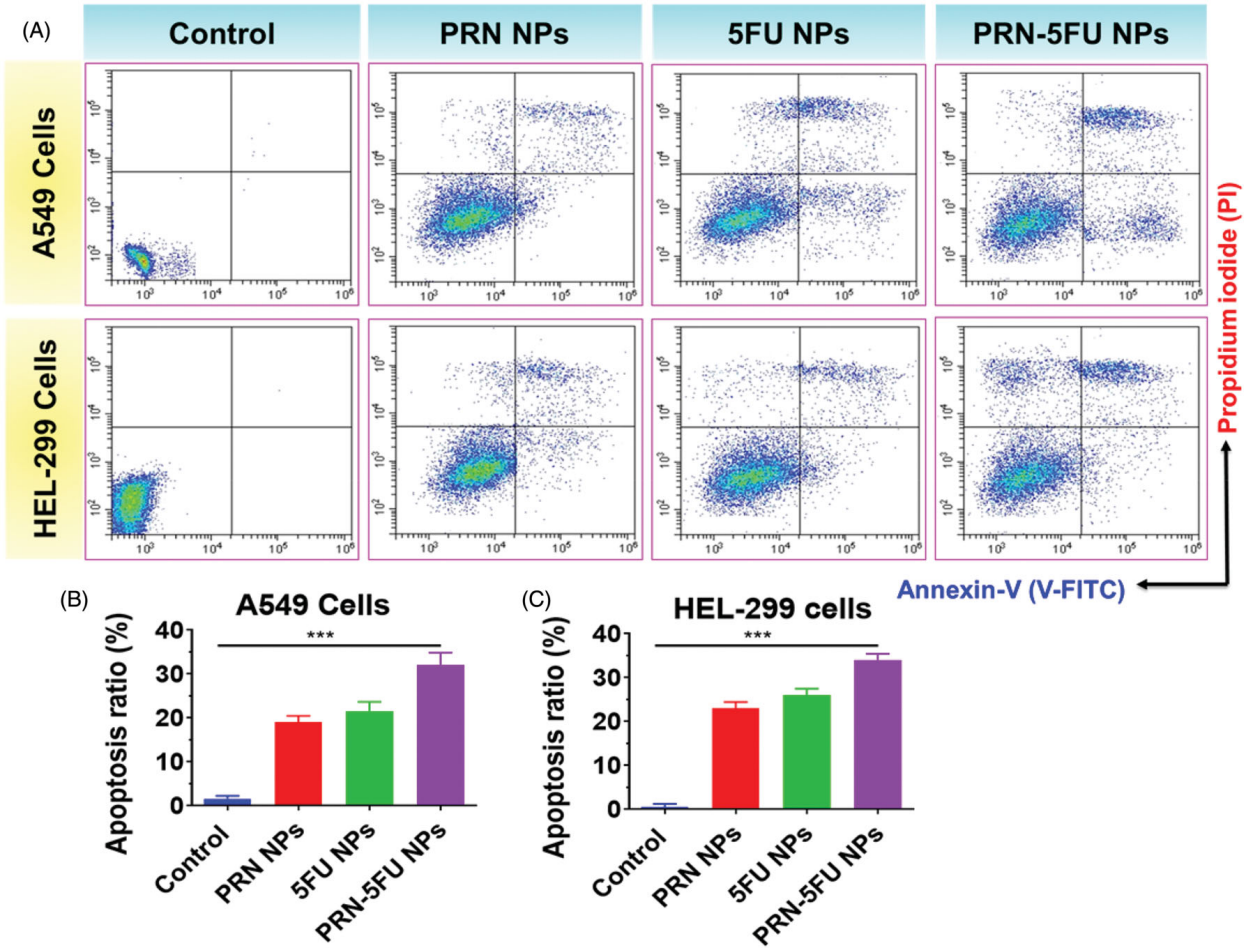
(A-C) apoptosis in human lung carcinoma cells (HEL-299 and A549) was confirmed using flow-cytometry analysis. Assessment of cell death in HEL-299 and A549 cancer cells treated with synthesized nanoparticles. HEL-299 and A549 cells were incubated at 37 °C for 24 h with different nanoparticles (control, 5FU NMs, PRN NMs, and PRN-5FU NMs) for 24 h.

To differentiate between normal, early, and late apoptotic cells, flow cytometric analysis with double staining was used. The apoptosis rate induced by 5FU NMs, PRN NMs, and 5FU-PRN NMs had an IC_50_ concentration, as shown in Figure 7(A–C). When compared to the control group, the overall proportion of apoptotic cells increased in the treatment group. According to these findings, 5FU NMs, PRN NMs, and 5FU-PRN NMs inhibited the development of HEL-299 and A549 cells by inducing apoptosis.

### 3.7. Hemolysis assay

**Figure 8.**
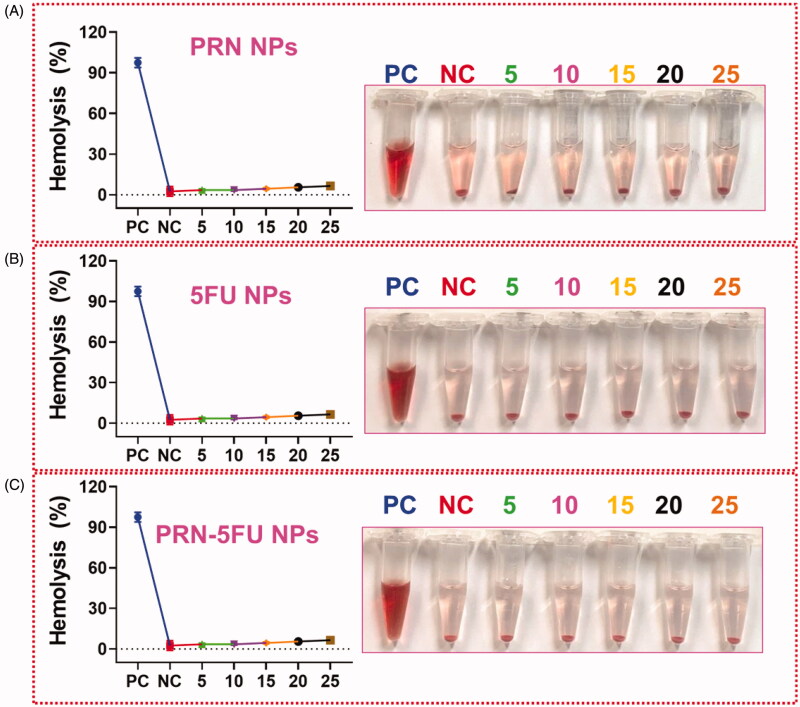
Hemolysis assay with different concentration of GEM and GEM-NMs. The result of hemolysis assay reveals that the insignificant hemolysis which shows that is extremely biocompatible for *in vivo* profiles.

NMs are known to interact with human red blood cells and cause hemolysis by disrupting the cell membrane. In vitro biocompatibility assay was used to investigate the impact of such an adverse effect on human health. The biocompatibility profiles of RBC induced by nanoparticles at different concentrations of 5–30 g/mL were demonstrated. The dose-dependent hemolytic effect shown in Figure 8 reduces the toxicity of 5FU NMs, PRN NMs, and 5FU-PRN NMs. According to the GEM and GEM-NMs results, we only observed minor hemolysis, indicating that it is highly biocompatible for *in vivo* profiles.

## 4. Conclusion

5FU-PRN NMs were constructed by immersing 5FU and PRN to improve drug aggregation and alter the tumor microenvironment. Finally, 5FU and PRN were successfully inserted into 5FU-PRN NMs through a through nanoassembling that included drug loadings and encapsulation. Based on this investigation, 5FU and PRN can be considered hydrophobic polymers based on the exercise of drug conveyance in oil/water solvents evaporation approaches. The 5FU-PRN NMs were then co-encapsulated in NMs with PRN and 5FU-centers coated with dioleoyl phosphatidic acids (DOPAs). The similarities of 5FU centers formed the essence of PRN into bio-degradable polymeric nanoparticles frameworks. The crystalline structure of the nanoparticles was also revealed using TEM electroscopic methods. PRN-5FU NMs, which included both 5FU and PRN, induced significant apoptosis in HEL-299 and A549 human lung cells. Dual staining techniques (AO/EB) and nucleus features were used to confirm the morphological findings in lung carcinoma cells (Hoechst 33258). The technology of flow cytometry was also used to investigate cell death pathways. These results may be used to refine anti-tumor models for lung carcinoma treatment, such as lipophilic and lipid-coated formulations.
